# Refinement of a clearing protocol to study crassinucellate ovules of the sugar beet (*Beta vulgaris* L., Amaranthaceae)

**DOI:** 10.1186/s13007-019-0452-6

**Published:** 2019-07-08

**Authors:** Monika Kwiatkowska, Dariusz Kadłuczka, Maria Wędzony, Beata Dedicova, Ewa Grzebelus

**Affiliations:** 1 Department of Plant Cytology and Embryology, Institute of Botany, Jagiellonian University, Gronostajowa 9, 30-387 Kraków, Poland; 2 Institute of Plant Biology and Biotechnology, Faculty of Biotechnology and Horticulture, University of Agriculture in Krakow, 29 Listopada 54, 31-425 Kraków, Poland; 3 Institute of Biology, Pedagogical University of Cracow, Podchorążych 2, 30-084 Kraków, Poland; 4 MariboHilleshög Research AB, Säbyholmsvägen 24, 261 91 Landskrona, Sweden

**Keywords:** Differential interference contrast (DIC), Embryogenesis, Megagametogenesis, Methyl salicylate, Plant embryology

## Abstract

**Background:**

Clearing methods allow relatively quick processing of plant material and examination of cellular structures by rendering tissues and organs translucent. They have been adapted for plant embryology, primarily to study ovule development, megasporogenesis, megagametogenesis and embryogenesis. Such clearing methods overcome several disadvantages of the conventional embedding-sectioning techniques that are arduous and time-consuming. Although numerous protocols with different clearing solutions have been described, there have been no reports to date proposing a reliable method to clear the crassinucellate ovules of the sugar beet (*Beta vulgaris* L.), an economically important crop. Therefore, this study aims to find a suitable approach to improve the tissue transparency of sugar beet ovules at different developmental stages.

**Results:**

We established a methyl salicylate-based protocol that significantly improved the transparency of the *B. vulgaris* ovule structures, which allowed us to observe the megagameto- and embryogenesis of that species. This was achieved by (1) chemical softening of the tissues; (2) vacuum pump-assisted infiltration step; (3) shaking-assisted incubation with clearing mixtures; and (4) manual removal of the chemically softened seed coat.

**Conclusions:**

The effectiveness of our method is due to the strategy combining various approaches at different stages of the procedure aiming at increasing the accessibility of the internal ovule structures to the clearing solution. The results of this study may be applied in sugar beet breeding programs, and it will provide a basis for further investigation of numerous aspects of the species’ embryology. Moreover, that unique approach may be easily adapted to other species developing crassinucellate ovules.

**Electronic supplementary material:**

The online version of this article (10.1186/s13007-019-0452-6) contains supplementary material, which is available to authorized users.

## Background

The sugar beet (*Beta vulgaris* L., Amaranthaceae) belongs to crops of great economic importance due to its storage root, which accumulates sucrose in high concentration. It is mostly grown for sugar production but it also serves as a source of bioethanol and a component of animal feed [1]. Developing new varieties is frequently based on homozygous lines which are produced by haploid/doubled haploid techniques [2]. Despite decades of studies, doubled haploid lines of sugar beet are obtained exclusively with one method, i.e. by gynogensis in vitro induction, in isolated ovule cultures. Haploids are induced at low frequency and their genome needs doubling before their practical application [3, 4]. An accessible and simple analysis of ovule structures could be a very valuable tool in precise and fast screening of induced in vitro gynogenesis, enabling fast evaluation of new induction protocols and evaluation of various genotype responses to new treatments.

The clearing technique is a well-known and appreciated method in plant embryology. It allows rapid observation of ovule structures at different stages of development including both sporophytic tissues and gametophytic cell lines. Since clearing protocols are relatively easy and enable reducing the number of steps in material preparation [5, 6], they are advantageous over the conventional embedding-sectioning procedures that are excessively laborious and time-consuming. The latter are especially tedious in research as they require analysis of numerous samples. Moreover, embedding-sectioning techniques make analysis difficult to interpret because three-dimensional structures are frequently distributed over several sections and thus a reconstruction of the full image is necessary [7, 8]. Nonetheless, chemical treatment—although effective for clearing—renders plant tissues and organs fragile. Therefore, spacers between the slide and the coverslip are often applied to avoid compressing the cleared sample [5, 9].

Over the years, clearing techniques have been used in various categories of plant research, especially in classical embryology. They were primarily applied to investigate the sexual reproduction of plants: in the analysis of anther development, microsporogenesis, microgametogenesis, pollen and pollen tube growth, ovule ontogeny, megasporogenesis, megagametogenesis, and embryogenesis [5, 10–14]. These methods are also important tools in the research on apomixis [6, 13, 15–18]. Additionally, clearing techniques are useful in studies on plant anatomy, e.g. on the structure of plant vascular system [19–21] and in experimental embryology, e.g. to analyze the development of embryos resulting from intergeneric crosses [22].

All clearing procedures are based on equilibration of the refractive index throughout the sample in order to reduce inhomogeneities in light scatter [23]. There are many clearing solutions that make maternal tissues translucent, enabling analysis of the gametophytic structures in pre-fertilization stages of the ovule development and the early sporophyte development [24]. One of the most commonly used clearing mixture was introduced by Herr [5]. It is composed of lactic acid, chloral hydrate, phenol, clove oil and xylene. Application of methyl salicylate as a clearing agent is another common approach introduced by Crane [25] and further modified by Young et al. [6]. Other procedures use various oxidative bleaches, such as hypochlorite [19], hydrogen peroxide [26], chlorine [27], and chromium trioxide [28]. Hoyer’s solution [29], lactophenol [30], dibutyl phthalate in combination with benzyl benzoate [15], Visicol™ [31], and more have been developed for various specific purposes. The result of a particular clearing method depends on its interaction with the studied plant tissue. Because tissue chemistry, cell sizes and their density all diverge from object to object and among different species, each clearing method should be carefully adjusted to the examined plant material. This aspect is especially important in embryology, where massive maternal tissue often surrounds generative cells, as observed in *B. vulgaris* ovules.

For decades, various aspects of beet embryology and reproductive biology have been investigated using embedding-sectioning techniques for light or electron microscopy. The aspects in question include ovule and embryo sac ultrastructure [32–34], gynogenic embryos development by in vitro techniques [2, 4, 35–41], seed development and germination [42] as well as apomictic embryo development through aposporic embryo sac formation, parthenogenesis or adventitious embryony [43]. However, the analysis of reproductive processes in the sugar beet has been very difficult due to the specific structure of its crassinucellate and bitegmic ovules including: (1) the formation of micropyle by the massive inner integument, (2) the development of the nucellar cap by the periclinal divisions of the apical cells in the nucellar epidermis, that elongate and become richly protoplasmic, (3) the formation of 5–6 layers of parietal cells above the sporogenous cell, (4) the deep position of the developing embryo sac in the nucellar tissue, (5) the outer epidermis of the testa—a mechanical layer with strongly thickened outer walls, saturated with tannins, (6) the thick seed coat formed by both integuments saturated with tannins, and (7) the exotestal arillate seed with the long curved embryo, surrounded by a thin layer of endosperm and a massive starchy perisperm [44–46], cited after [47, 48]. Moreover, during the flower development, ovules keep changing their position, which hinders the proper orientation of the specimen during conventional embedding in the supporting matrices. As a consequence, a large amount of improperly cut ovules cannot be correctly analyzed, which results in substantial material losses. It should be emphasized that such difficulties do not occur in the clearing of tenuinucellate ovules, as exemplified by the model plant *Arabidopsis thaliana* [49–51], *Rudbeckia bicolor* [52], *Taraxacum atricapillum* [53] as well as in other crassinucellate ovules, especially those with a relatively small amount of nucellus cells, e.g. *Agave tequilana* [54], *Cenchrus ciliaris* [6], *Paspalum rufum* [55], *Ranunculus auricomus* [56]. Therefore, here for the first time, we report a successful methyl salicylate-based method for clearing of crassinucellate sugar beet ovules at different stages of their development allowing unambiguous visualization of ovule structures, the gametophyte and the embryo development. We believe, that this protocol may also be helpful in embryological studies on other crop species with the same or similar anatomical ovule structure, such as *Chenopodium quinoa* [57], *Papaver somniferum* [58], *Spinacia oleracea* [59], *Amaranthus caudatus*—called the crop of the XXI century [60], and others.

## Methods

### Plant material

To improve the clearing procedure, ovules of *Beta vulgaris* (breeding lines L365 and LY64, Syngenta Seeds AB, Sweden) at different stages of development were used. Additionally, for comparison purposes, tenuinucellate ovules of the model plant *Arabidopsis thaliana* L. and *Biscutella laevigata* L. as well as other crassinucellate ovules of *Armeria maritima* (Milld.) Willd. and *Viola banksii* K.R. Thiele & Prober were subjected to the same clearing protocol.

The flowers of *B. vulgaris* and *A. thaliana* were collected from seed-produced plants. The seeds of *B. vulgaris* were treated against seed borne diseases by 5-min incubation in 57 °C water bath before planting into sowing soil Emmaljunga I (Emmaljunga Torvmull AB, Sweden). The seedlings were grown in 1-L pots in a climate chamber at 18/12 °C (day/night) with a 22-h photoperiod in the light with the intensity of 90 µmol m^2^ s^−1^, with additional CO_2_ enrichment at the concentration of 400 ppm and 60–70% of relative humidity. After 3–4 weeks the plants with 6–8 leaves were transferred into a vernalization chamber at 6–9 °C, with a 12-h photoperiod in the light with the intensity of 70 µmol m^2^ s^−1^. After a 14-week vernalization period, well developed plants with 8–10 leaves were re-planted into 2-L pots with planting soil Emmaljunga II (Emmaljunga Torvmull AB, Sweden) and grown in a climate chamber at 18/16 °C (day/night) with a 18-h photoperiod in the light with the intensity of 480–500 µmol m^2^ s^−1^ (metal halide lamp bulbs 400 W GE Kolorarc, Hungary), with additional CO_2_ enrichment at the concentration of 600 ppm and 60–70% of relative humidity. Approximately 6–8 weeks later, the flowers were collected.

The seeds of *A. thaliana* were sown to a moss-sandy substrate (Hollas, Poland) and incubated in a climate room at 20 ± 2 °C with a 16-h photoperiod in the light with the intensity of 40 µmol m^−2^ s^−1^ for germination and plant growth. About 10 weeks later, the flowers of *A. thaliana* were collected.

The flowers of *B. laevigata* and *A. maritima* were collected from their natural habitat in Bolesław (Poland), while the flowers of *V. banksii* were obtained from the collection of the Department of Plant Cytology and Embryology, Jagiellonian University (Krakow, Poland).

### Ovule treatment and image acquisition

All flowers were fixed in FAA (38% formaldehyde/glacial acetic acid/70% ethanol; 6:4:90, v/v) for 48 h, and stored in 70% ethanol in 4 °C until further use. Ovules at different developmental stages, in ovaries or dissected from ovaries, were dehydrated in a graded ethanol series (70%, 95%, 100%—1 h each). After the 95% ethanol solution, the ovules in pre-fertilization stages were incubated in eosin (2% solution in 95% ethanol, w/v) for 1 h to improve their visibility during further processing. Then the ovules were cleared initially with methyl salicylate, according to Young et al. [6], with minor modifications after Musiał et al. [53], involving an additional step of treatment with the clearing solution, and modified incubation time. In detail, the infiltration procedure was performed as follows: (1) the ovules were treated with a mixture of 100% ethanol and methyl salicylate in proportions of 3:1, 1:1, 1:3 (1.5 h each), and then (2) incubated with pure methyl salicylate for 24 h—hereinafter referred to as the standard procedure—or (3) with prolonged incubation time, of up to 4 weeks (Table 1). In order to improve the tissue transparency in *B. vulgaris* ovules, numerous modifications of the standard procedure were tested, including the use of macerating agents (such as inorganic acids of different concentrations, i.e. 0.1 or 1 M HCl, 3 or 95% H_2_SO_4_, 1% H_5_IO_6_), and other chemicals, such as Schiff’s reagent consisting of 1% (w/v) basic fuchsin and 2,3% (w/v) K_2_S_2_O_5_ in 0.15 M HCl, and 3 or 6% (v/v) hydrogen peroxide—both separately and combined. The manipulation of the incubation time of the material in the solutions was combined with an application of the vacuum pump (Concentrator plus, Eppendorf, Hamburg, Germany) and orbital shaker (150 rpm), or mechanical tissue disruption using a syringe needle, all in order to improve tissue penetration by the clearing solution. All the steps in these modified procedures and the remarks on them are shown in detail in Additional file 1: Table S1. The best approaches were selected and applied to further studies (Table 2). The cleared ovules were prepared on microscope slides under a dissecting microscope, in a drop of methyl salicylate, according to Herr [5]. The slides were examined using differential interference contrast (DIC) optics under an Axio Imager.M2 microscope (Carl Zeiss, Göttingen, Germany) equipped with an AxioCam MRm camera (Carl Zeiss, Göttingen, Germany), and processed with AxioVision 4.8 (Carl Zeiss MicroImaging) and Adobe^®^ Photoshop^®^ CS3 (Adobe Systems) software. Over 15 ovules per protocol variant were examined. The ovule structure as well as megagametogenesis and embryogenesis were analyzed.

**Table 1:** Clearing effects of methyl salicylate on exemplary tenuinucellate and crassinucellate ovules in pre- and post-fertilization stages using the standard^a^ procedure or its modified^b^ version (n ≥ 15)

Ovule type	Species	Clearing procedure	Clearing effect^c^
Tenuinucellate	*Arabidopsis thaliana* ^d^	Standard	+
		Modified	++
Crassinucellate	*Biscutella laevigata*	Standard	+
	*Armeria maritima*	Standard	+
	*Viola banksii*	Standard	+
	*Beta vulgaris*	Standard	−
		Modified	−

^a^The standard procedure: absolute ethanol : methyl salicylate, in proportions of 3:1, 1:1, 1:3, 0:1 (1.5 h each change)

^b^The modified procedure: as the standard, except the step of clearing in pure methyl salicylate, which was prolonged from 24 h to 4 weeks

^c^(++) very well cleared ovules; (+) ovules cleared at an acceptable level; (−) ovules cleared at an unsatisfactory level

^d^The whole ovaries were cleared to prevent the material loss during its preparing process

**Table 2:** The optimized protocols for improved clearing efficacy in crassinucellate ovules of *B. vulgaris* (n ≥ 15)

Ovule development	Tissue processing		Notes
	Treatment	Conditions^a^	
Pre-fertilization stages^b^ (protocol I)	Rehydration^c^	On an orbital shaker^d^	1, 2, 3
	1 M HCl	5 min	
	1 M HCl	60 °C, 10 min	
	1 M HCl	5 min	
	Schiff’s reagent	30 min, in the dark	
	Sulfur water^e^	3 × 10 min	
	*d*H_2_O	3 × 5 min	
	Dehydration^f^	On an orbital shaker	
	Clearing^g^	On an orbital shaker, with vacuum treatment^h^	
Post-fertilization stages (protocol II)	Rehydration	On an orbital shaker	2, 4
	95% H_2_SO_4_	5 min	
	*d*H_2_O	5 min	
	1 M HCl	5 min	
	1 M HCl	60 °C, 10 min	
	1 M HCl	5 min	
	Schiff’s reagent	30 min	
	Sulfur water	3 × 10 min	
	*d*H_2_O	3 × 5 min	
	Dehydration	On an orbital shaker	
	Clearing	On an orbital shaker, with vacuum treatment	
	Manual removal of the seed coat		
Post-fertilization stages (protocol III)	Rehydration	On an orbital shaker	4, 5, 6
	95% H_2_SO_4_	5 min	
	*d*H_2_O	5 min	
	3% H_2_SO_4_	60 °C, 90 min	
	*d*H_2_O	3 × 5 min	
	Dehydration	On an orbital shaker	
	Clearing	On an orbital shaker, with vacuum treatment	
	Manual removal of the seed coat		

1. The tissue transparency was significantly improved

2. Schiff’s reagent stained ovules making the tissues visible—eosin treatment may be omitted

3. Longer than 24-h incubation in pure methyl salicylate improved the tissue transparency

4. The tissue transparency was improved due to the removable seed coat, caused by the use of H_2_SO_4_

5. In the case of ovules in pre-fertilization stages, maceration in concentrated sulfuric acid may be omitted

6. Protocols II and III may be used interchangeably, since both gave the same clearing results

^a^All the steps were performed at room temperature, unless otherwise specified

^b^The whole ovaries were cleared to prevent the material loss during its preparation process

^c^Rehydration = ethanol: 70%, 50%, 30%; *d*H_2_O (5 min each)

^d^Parameters of shaking were set at 150 rpm

^e^Sulfur water was made with 5 mg mL^−1^ K_2_S_2_O_5_ in 0.05 M HCl

^f^Dehydration = ethanol: 10%, 30%, 50%, 70% (15 min each); 95%, 100% (1 h each)

^g^Clearing = 100% ethanol: methyl salicylate, in proportions of 3:1, 1:1, 1:3 (2 h each change); pure methyl salicylate (at least 24 h)

^h^After 1 h of each change of the clearing solution, vacuum treatment for 5 min was applied

## Results

**Fig. 1:**
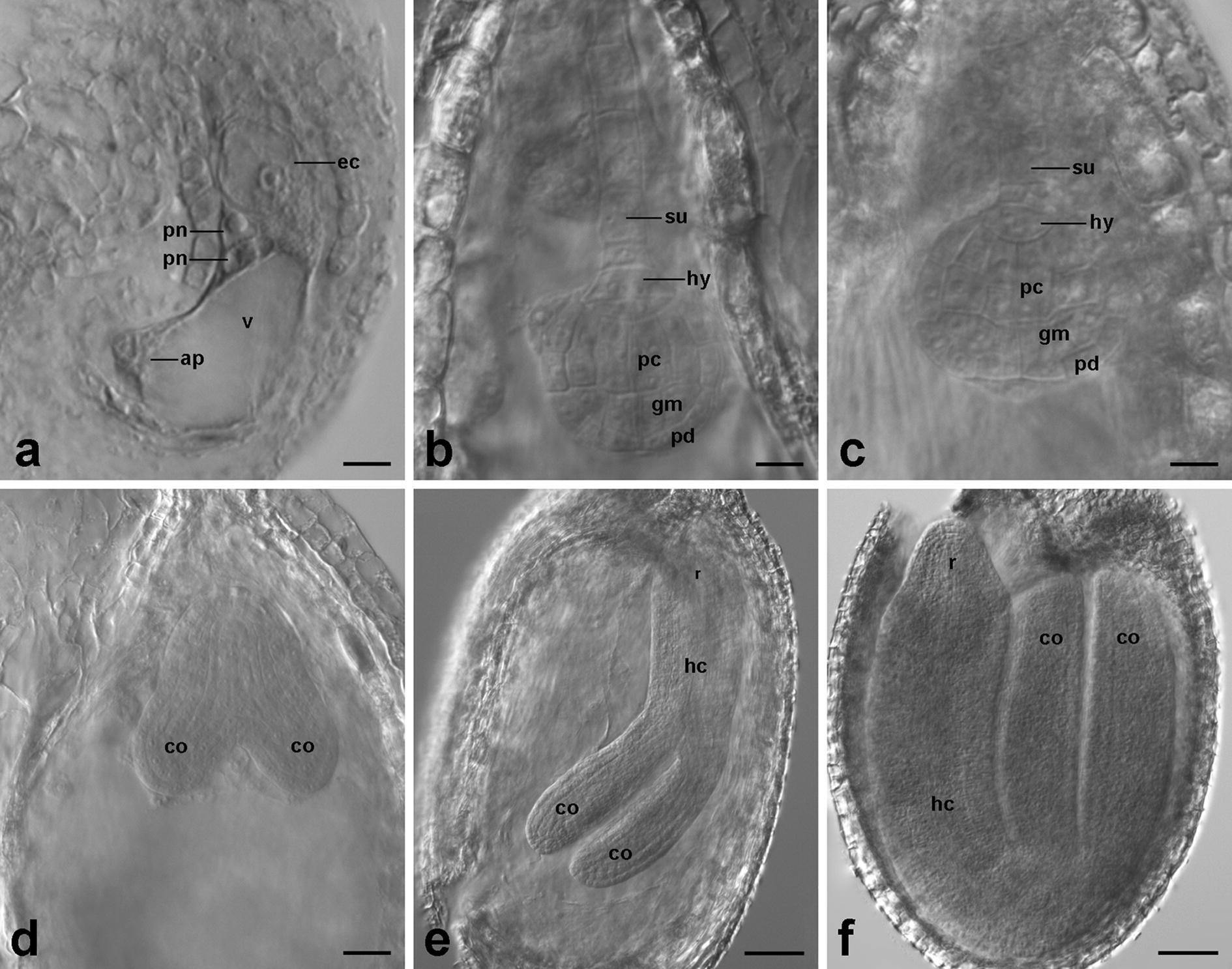
Differential interference contrast (DIC) images of *Arabidopsis thaliana* ovules cleared by the standard procedure with prolonged incubation time in pure methyl salicylate up to 4 weeks. **a** Mature embryo sac with a central cell before polar nuclei fusion. **b–f** Subsequent stages of embryogenesis. **b** Globular-shaped embryo. **c** Late globular-shaped embryo. The bilateral symmetry of the proper embryo begins to form. **d** Late heart-shaped embryo. **e** Walking-stick-shaped embryo. **f** Mature embryo. *ap*, antipodal cells; *co*, cotyledon; *ec*, egg cell; *gm*, ground meristem; *hc*, hypocotyl; *hy*, hypophysis; *pc*, procambium; *pd*, protoderm; *pn*, polar nucleus in the central cell; *r*, radicle; *su*, suspensor; *v*, vacuole. Scale bars: 10 µm (**a–c**), 20 µm (**d**), 50 µm (**e**, **f**)

**Fig. 2:**
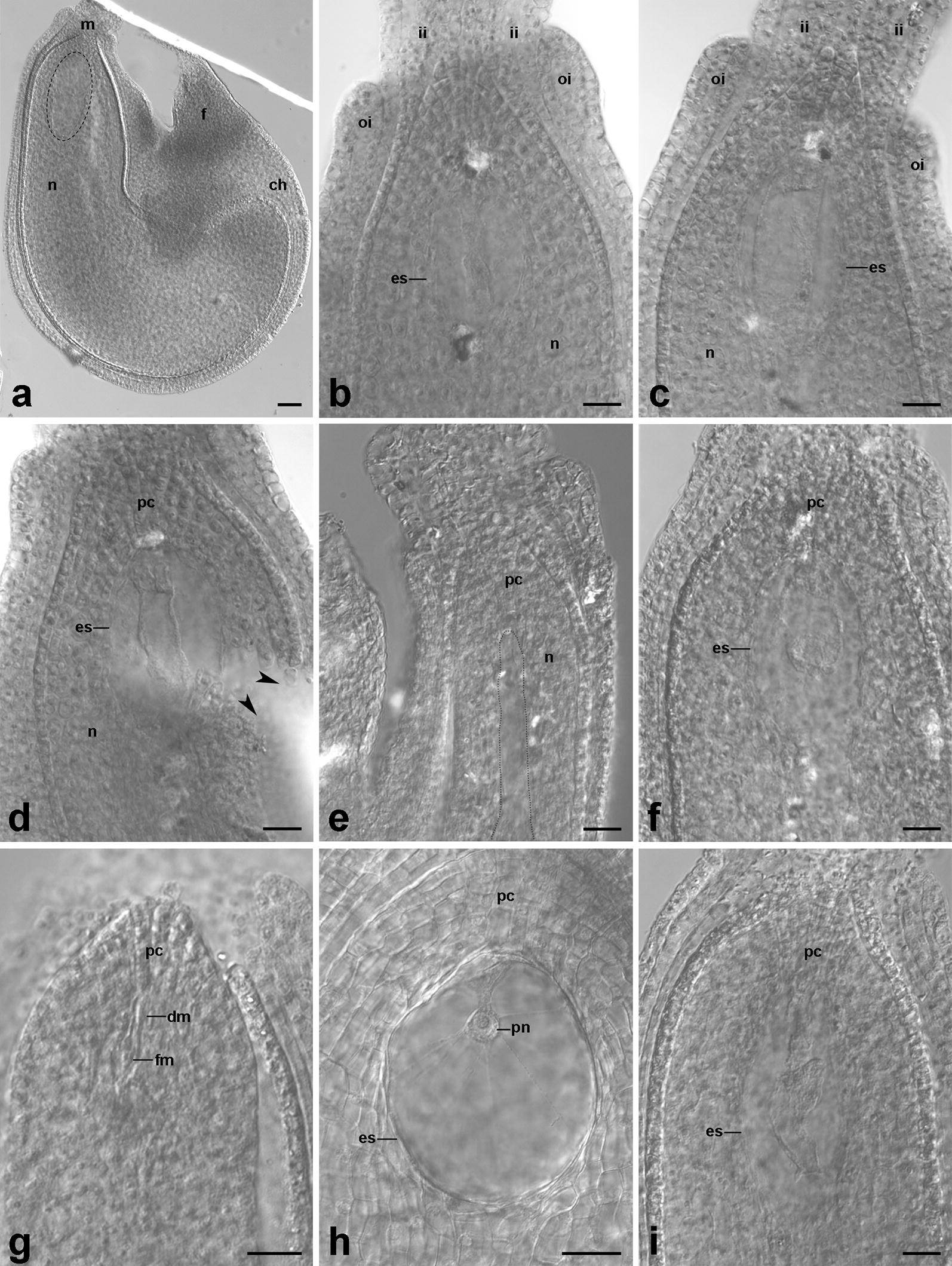
Differential interference contrast (DIC) images of *Beta vulgaris* ovules cleared by the standard procedure with methyl salicylate (**a**), and the exemplary effects of its modifications tested (**b–i**). **a** Ovule with hardly visible embryo sac (*dotted line*). **b** Micropylar pole of the ovule with a mature embryo sac after the standard procedure with prolonged incubation time in pure methyl salicylate for up to 4 weeks. **c** Ovule with a mature embryo sac subjected to shaking and vacuuming during infiltration steps. **d** Needle-disrupted ovule (*arrowheads*) with a mature embryo sac. **e** Over-macerated ovule with constricted embryo sac after pre-treatment with 6% hydrogen peroxide. **f** Well cleared ovule after pre-treatment with 3% sulfuric acid. Image quality distorted by light reflection. **g–i** Well cleared ovules after pre-treatment with 0.1 M hydrochloric acid combined with Schiff’s reagent. **g** Ovule after meiosis with a functional megaspore formed. **h** Mature embryo sac with visible polar nucleus of the central cell. **i** Mature embryo sac. c*h*, chalazal pole of the ovule; *dm*, degenerated megaspore; *es*, embryo sac; *f*, funiculus; *fm*, functional megaspore; *ii*, inner integument; *m*, micropylar pole of the ovule; *n*, nucellus; *oi*, outer integument; *pc*, parietal cells; *pn*, polar nucleus of the central cell. Scale bars: 20 µm (**b–i**), 50 µm (**a**)

The standard clearing procedure applied to the tenuinucellate ovules of *Arabidopsis thaliana* and *Biscutella laevigata* resulted in a satisfactory level of their structures’ transparency (Table 1), as observed for all the analyzed ovules. The application of eosin before tissue clearing improved the visibility of small structures, preventing material loss during preparation, while it did not negatively interfere with microscopic observations. Regardless of the developmental stages of the examined ovules, the tissues were very well cleared, and both sporophytic and gametophytic cells were clearly visible (Additional file 2: Fig. S1; Additional file 3: Fig. S2b). The only exceptions were *A. thaliana* ovules at globular- and heart-shaped embryo stages, where the developing seed coat somewhat hampered the penetration of methyl salicylate causing insufficient tissue transparency (Additional file 2: Fig. S1d). In this case, the increase in the incubation time in pure methyl salicylate to as many as 4 weeks contributed to improved transparency of the ovule structures which were better cleared, compared to the standard procedure (Fig. 1). The standard procedure on the crassinucellate ovules of *Armeria maritima* and *Viola banksii* resulted in effectively cleared embryo sac cells in all the analyzed ovules (Table 1; Additional file 3: Fig. S2a, c). Conversely, this procedure was unsuccessful for the crassinucellate ovules of *Beta vulgaris*, both in early and late stages of development, resulting in tissues that were not transparent enough to make observations. The visibility of the generative cells was unsatisfactory, and the female gametophyte was only slightly visible (Fig. 2a). Also, the prolonged time of the methyl salicylate treatment did not result in any improvement in the clearing of *B. vulgaris* ovules (Fig. 2b).

All other modifications and the remarks on them are listed in Additional file 1: Table S1, showing the detailed procedures which were tested on sugar beet ovules in the present study. Among these, mechanical tissue disruption using a syringe needle, aimed at opening the ovule structure and facilitating penetration of the clearing mixtures did not succeed due to the small size of the ovules and an increased risk of damaging the female gametophyte. Moreover, this treatment resulted in low-quality images (Fig. 2d). Other attempts were based on the use of post-clearing treatments with some macerating agents (3% hydrogen peroxide, 3% sulfuric acid), but they did not improve the transparency of the ovule tissues. Furthermore, several chemicals were used before the clearing step, such as 1% orthoperiodic acid, 0.1 M hydrochloric acid, both combined with Schiff’s reagent, 6% hydrogen peroxide and 3% sulfuric acid (Fig. 2e–i), of which only the application of the hydrochloric acid with Schiff’s reagent systematically brought better results, exclusively in the case of young ovules (Fig. 2h). The application of shaking and vacuuming treatment during infiltration steps was another promising variant regularly improving the transparency of the ovule tissue (Fig. 2c).

**Fig. 3:**
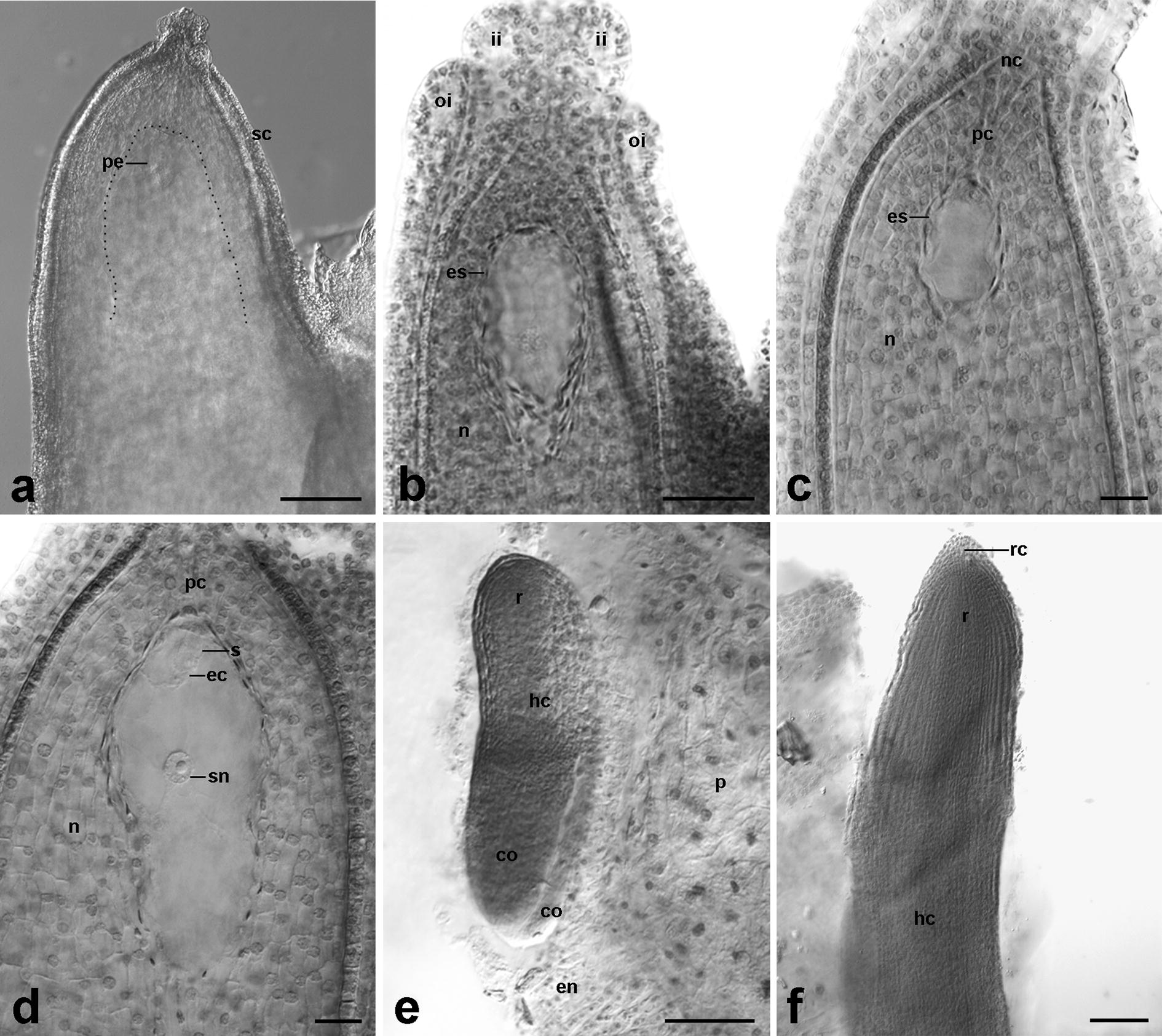
Differential interference contrast (DIC) images of *Beta vulgaris* ovules cleared by the modified procedure. The material was pre-treated with 1 M hydrochloric acid combined with Schiff’s reagent, and subjected to shaking and vacuuming during infiltration steps. **a** Ovule at post-fertilization stage. The proembryo in the embryo sac slightly visible due to the presence of the developing seed coat (negative control). **b–f** Ovules after optimization of the modified procedure. **b** Ovule with an embryo sac. **c** Micropylar pole of the ovule with a young embryo sac. **d** Ovule with a mature embryo sac after polar nuclei fusion. **e**, **f** Before the maceration step with hydrochloric acid, the material was pre-treated with 95% sulfuric acid in order to manually remove the seed coat. **e** Torpedo-shaped embryo. **f** Apical part of a mature embryo with embryonic tissues of hypocotyl and radicle surrounded by a root cap. **b–d** Pre-fertilization stages; **a**, **e**, **f** post-fertilization stages. *Black dotted line* surrounds an embryo sac. *co*, cotyledon; *ec*, egg cell; *en*, cellular endosperm; *es*, embryo sac; *hc*, hypocotyl; *ii*, inner integument; *n*, nucellus; *nc*, nucellar cap; *oi*, outer integument; *p*, perisperm; *pc*, parietal cells; *pe*, proembryo; *r*, radicle; *rc*, root cap; *s*, synergid; *sc*, seed coat; *sn*, secondary nucleus of the central cell. Scale bars: 20 µm (**c**, **d**), 50 µm (**b**), 100 µm (**a**, **e**, **f**)

**Fig. 4:**
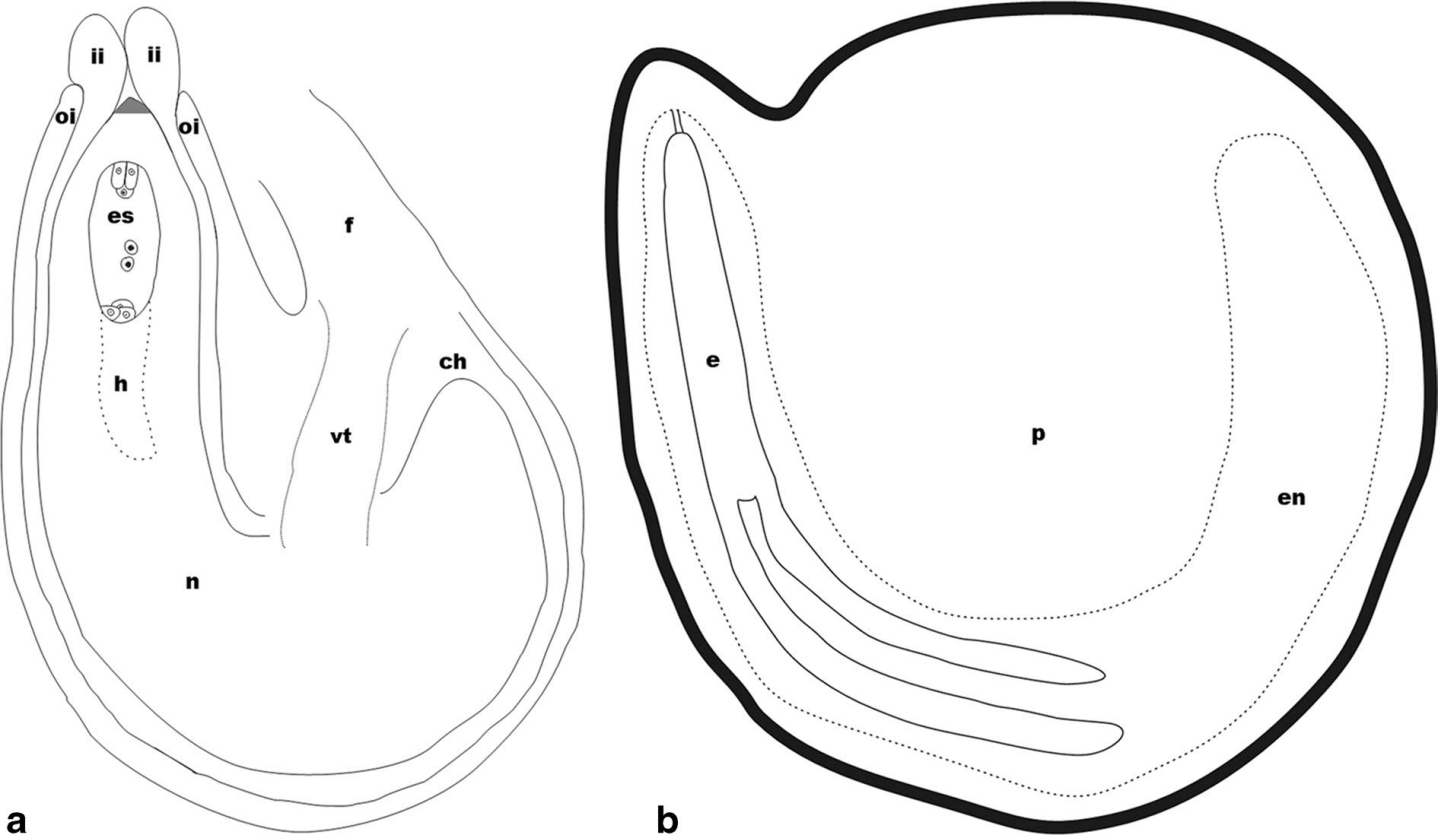
Schematic diagram depicting the structure of the crassinucellate ovules of *Beta vulgaris* at different developmental stages. **a** Ovule at pre-fertilization stage with a mature embryo sac; the dotted line indicates a haustorium (*h*), originating from the central cell of the embryo sac; the grey area represents a nucellar cap on the micropylar pole of the ovule. **b** Ovule at post-fertilization stage with a growing embryo; the bold line represents the developing seed coat; the dotted line indicates the border of the embryo sac. *ch*, chalazal pole of the ovule; *e*, embryo with growing cotyledons; *en*, cellular endosperm; *es*, embryo sac; *f*, funiculus; *ii*, inner integument; *n*, nucellus; *oi*, outer integument; *p*, massive perisperm; *vt*, vascular tissue

From the modifications of the standard procedure, we selected those that significantly improved the transparency of *B. vulgaris* ovule structures (Table 2, whereby the procedures were divided based on the stage of ovule development into pre- and post-fertilization stages, which was accompanied by the absence or presence of the seed coat, respectively). Such an improvement was achieved by novel, never previously applied, combinations of various approaches at different stages of the procedure. Firstly, the use of vacuum pump to infiltrate the material with methyl salicylate mixtures, and the fact that the clearing step was performed with continuous shaking, rendered the penetration of the relatively dense clearing solution much more effective. Secondly, both the ovules before fertilization and the developing seeds were treated with hydrochloric acid (1 M HCl) in 60 °C combined with Schiff’s reagent, causing their softening. Thirdly, the ovules in post-fertilization stages (developing seeds) were pre-treated with highly concentrated sulfuric acid (95% H_2_SO_4_), which made it possible to remove the seed coats, increasing the accessibility of the internal tissues to the clearing solution. If concentrated sulfuric acid was not used, even at very young stages of embryogenesis, the developing hard seed coat hindered the clarity and transparency of ovule tissues, even after applying maceration procedure before clearing (Fig. 3a). Thus, by applying these combined approaches, *B. vulgaris* ovule tissues systematically showed a satisfactory level of transparency, allowing observation of the developing embryo sacs and embryos (Fig. 3b–f). Additionally, the material hydrolyzed by hydrochloric acid was exposed to further reaction with Schiff’s reagent, which stained it reddish pink, preventing the loss of small objects. For clarity, a schematic diagram illustrating the structure of the crassinucellate ovules of *B. vulgaris* at pre- and post-fertilization stages is included in Fig. 4.

## Discussion

As the rapid development of microscopy techniques over the years has advanced, the ability to visualize plant tissues and cell structures has greatly increased. However, plant features limit their transparency to visible light, which prevents access to their internal structures [9]. Therefore, with the aim of obtaining images of better quality, numerous clearing protocols that render plant tissues or organs translucent without disturbing their anatomy have been described, of which the use of methyl salicylate as a clearing agent is very common for studying ovules (e.g. [6, 7, 22, 52, 61]. The clearing technique is a quick and simple method frequently used in classical embryology to analyze ovule development, embryo sac formation, embryogenesis, and seed development [5, 6, 13, 16, 17]. Moreover, it requires less effort than conventional embedding-sectioning procedures, that are arduous and time-consuming [7, 8].

We showed that the standard clearing procedure with methyl salicylate applied, according to Young et al. [6], with minor modifications by Musiał et al. [53], was an appropriate method to make the tenuinucellate ovules of *A. thaliana* and *B. laevigata* transparent. It was also suitable for clearing the crassinucellate ovules of *A. maritima* and *V. banksii*. However, this approach was insufficient for the crassinucellate ovules of *B. vulgaris*, where female gametophytes were only slightly visible after processing. This was due to the specific structure of sugar beet ovules, i.e. the embryo sac location deep in the thick nucellar tissue surrounded by parietal cells and massive, multi-layered integuments, and by the presence of a nucellar cap at the micropylar pole. Moreover, in developing seeds, integuments form a hard and dark seed coat, that is rich in tannins, and a starchy perisperm occurs that disturbs analyses, as reported previously [32–34, 44, 47, 48, 62]. The abovementioned features caused difficulties in penetration of the clearing mixtures into the internal tissues of the ovules. Hence, the search for a new protocol to clear the *B. vulgaris* ovules was necessary to enable a large-scale screening of developing ovules and seeds.

To date, embryological observations in the sugar beet have been conducted mostly by means of embedding-sectioning techniques [32, 33, 36, 38, 63]. To our knowledge, the only report published to date which addresses the use of clearing solution to observe sugar beet ovules is that by Ferrant and Bouharmont [38], who applied a modified stain-clearing technique by Stelly et al. [8] that involved Mayer’s hemalum and methyl salicylate. However, the authors applied the clearing technique as complementary to the primary conventional one, which includes embedding, cutting and staining the material, which renders it a multi-step and, therefore, time-consuming approach, especially when analyzing many samples in a short time. Thus, unlike our protocol, their method is not suitable for large scale studies. Bearing in mind all the previous attempts, we aimed to apply the methyl salicylate-based clearing technique as the basic one to characterize a large amount of *B. vulgaris* ovules at different developmental stages. We have added numerous variants of the method that allowed us to select the best protocols for young ovules and for later stages of seed development in *B. vulgaris*.

In current literature, the clearing technique is routinely used in research on the ovule and seed development of the model plant *A. thaliana,* especially in the research on expression and in situ localization of genes in various mutants. In these studies, chloral hydrate, which is known to be toxic, is often used as the clearing agent (e.g. [49–51, 64]. We, therefore, suggest using methyl salicylate instead, which brought about good clearing results (Table 1; Additional file 2: Fig. S1). As we can conclude from our experiments, an even higher level of clarity is achieved by simply extending the incubation period in pure methyl salicylate (Fig. 1). Contemporary research on embryological processes, including observations of classical sexual reproduction stages as well as apomictic processes has been widely conducted using the clearing technique with methyl salicylate, as, for instance, in *Rudbeckia bicolor* [52], *Agave tequilana* [54], or *Ranunculus auricomus* [56]. Additionally, our observations also indicate that methyl salicylate may be applied to clear effectively the tennuinucellate ovules of *B. laevigata*, and the crassinucellate ovules of *A. maritima* and *V. banksii*.

Clearing crassinucellate ovules has always been problematic, not only because of the thick layers of the nucellus tissue, but also because of its starchy content, thick integuments and early seed coat formation. The study on apomixis in cassava (*Manihot esculenta* Cranz) is a good example of the problem [65]. The authors published molecular data (analysis with RAPD markers) along with anatomic studies to prove apomixis in this species, since pictures of the anatomic analyses conducted by clearing were not convincing. This case brings about another problem: the proper equipment to visualize the cleared objects and their proper documentation. When dealing with cleared whole-mount ovules, differential interference contrast (DIC) microscopy is a useful tool for observing their internal structures, as shown in this paper. However, some works in the literature suggest that a whole-mount eosin B-staining confocal laser scanning microscopy (WE-CLSM) may also be a promising method worth considering in future studies [66, 67]. This approach is, moreover, also applicable with methyl salicylate, as documented by these authors, who used it for investigating abnormal embryo sacs in *indica/japonica* rice hybrids, as well as in similar studies by other authors (e.g. [68, 69]).

After testing numerous modifications of the standard procedure, we established the most suitable approach for clearing the crassinucellate ovules of *B. vulgaris* at several developmental stages. Most of the modifications in the development of our protocol aimed to increase the accessibility of internal ovule structures to the clearing solution (methyl salicylate), which was obtained by (1) chemical softening of the tissues, (2) the use of a vacuum pump in order to infiltrate the internal ovule tissues with methyl salicylate, (3) the application of continuous gentle shaking throughout the incubation step with clearing mixtures, and (4) manual removal of the chemically softened seed coat. The results show the effectiveness of our method, making it possible to study the megagameto- and embryogenesis of *B. vulgaris* (Fig. 3b–f). The proposed protocol can be directly applied both to research and breeding programs to evaluate the development of gynogenic embryos in that species and thus to improve the breeding process of the crop of great economic importance.

## Conclusions

In this study, we established an improved protocol for clearing the crassinucellate ovules of *B. vulgaris*, relying mainly on a novel combination of various pre-treatments increasing the accessibility of the tissue to methyl salicylate, which allowed us to investigate female gametophyte of the sugar beet and its embryo development. Many laboratories could substantially benefit from the results of our experiments. Especially the laboratories which rely on the routine microscopic analysis of sugar beet ovules, particularly while developing new protocols for haploid production. The presented clearing protocol may also provide a useful tool for further investigations on the flowering biology of the sugar beet and other species developing crassinucellate ovules, e.g. the cucumber, the evening primrose and others.

## Additional files

**Additional file 1: Table S1.** Attempts to modify the standard clearing procedure applied to improve the transparency of the *B. vulgaris* ovules.

**Additional file 2: Fig. S1.** Differential interference contrast (DIC) images of *Arabidopsis thaliana* ovules in subsequent stages of development cleared by the standard procedure with methyl salicylate. (**a**) Fragment of an ovary with multiple ovules. (**b**) Young ovule with two-nucleate embryo sac. (**c**) Ovule with mature embryo sac. (**d**) Early torpedo-shaped embryo. (**e**) Late torpedo-shaped embryo. (**f**) Mature embryo. c*h*, chalazal pole of the ovule; *co*, cotyledon; *ec*, egg cell; *en*, cellular endosperm; *ep*, epidermis; *es*, embryo sac; *hc*, hypocotyl; *ii*, inner integument; *oi*, outer integument; *p*, plumule; *r*, radicle; *sc*, seed coat; *sn*, secondary nucleus of the central cell; *su*, suspensor. Scale bars: 10 µm (**b, c**), 20 µm (**d**), 50 µm (**a**, **e, f**).

**Additional file 3: Fig. S2.** Differential interference contrast (DIC) images of *Armeria maritima* (**a**), *Biscutella laevigata* (**b**), and *Viola banksii* (**c**) ovules cleared by the standard procedure with methyl salicylate. *ap*, antipodal cells; *ea*, egg apparatus; *ec*, egg cell; *ii*, inner integument; *n*, nucellus; *oi*, outer integument; *pn*, polar nucleus; s, synergid; *sn*, secondary nucleus of the central cell. Scale bars: 20 µm (**b**), 50 µm (**a**, **c**).

## Data Availability

All data generated or analyzed during this study are included in this published article and its supplementary information files.
