# Supplementary material for: Refinement of a clearing protocol to study crassinucellate ovules of the sugar beet (Beta vulgaris L., Amaranthaceae)
Source: Plant Methods. 2019 Jul 8;15:71. doi: 10.1186/s13007-019-0452-6 (PMC6613245; doi:10.1186/s13007-019-0452-6)
Supplement: Supplementary file 1 — Additional file 1: Table S1. Attempts to modify the standard clearing procedure applied to improve the transparency of the B. vulgaris ovules. [file 13007_2019_452_MOESM1_ESM.docx]

**Additional file 1**: **Table S1**. Attempts to modify the standard clearing procedure applied to improve the transparency of the *B. vulgaris* ovules

| Ex. No.^a^ | Tissue processing | | Comments |
| --- | --- | --- | --- |
|  | Treatment | Conditions^b^ |  |
| 1. | dehydration^c^  clearing^d^ | | – In younger ovules, the tissue transparency was improved, compared to the standard procedure.  – In older ovules, the tissue transparency was not improved due to the presence of the massive integuments. |
| 2. | dehydration  clearing  punctures of cleared ovules^e^ | | – The risk of damage to the ovule structures was increased.  – Low-quality images were obtained. |
| 3. | dehydration  70% ethanol  2% eosin^f^  95% ethanol  100% ethanol  clearing | 15 min  1 h  1 h  1 h | – Visibility of the ovules was improved.  – Eosin staining prevented the loss of the material during its preparation.  – Procedure did not interfere with microscopic observations. |
| 4. | dehydration  clearing | | – The tissue transparency was not improved. |
|  | 3% H_2_O_2_ | 24 h, directly on slides |  |
| 5. | dehydration  clearing | | – The tissue transparency was not improved. |
|  | 3% H_2_SO_4_ | 24 h, directly on slides |  |
| 6. | *d*H_2_O  1% H_5_IO_6_  Schiff’s reagent  sulfur water^g^  tap water  *d*H_2_O | 5 min  60°C, 10 min  30 min, in the dark  3× 10 min  30 s  5 min | – The tissue transparency was not improved.  – Both integuments were too darkly purple stained to allow proper visualization of ovule internal structures. |
|  | dehydration II^h^  clearing | |  |
| 7. | 95% ethanol  *d*H_2_O  6% H_2_O_2_  tap water  *d*H_2_O | 15 min  5 min  60°C, 1 h  30 s  5 min | – Tissues were over-macerated. |
|  | dehydration II  clearing | |  |
| 8. | *d*H_2_O  0.1 M HCl  0.1 M HCl  0.1 M HCl  Schiff’s reagent  sulfur water  tap water  *d*H_2_O | 5 min  5 min  60°C, 10 min  5 min  30 min, in the dark  3× 10 min  30 s  5 min | – In younger ovules, the tissue transparency was improved.  – In older ovules (developing seeds), the tissue transparency was not improved. |
|  | dehydration II  clearing | |  |
| 9. | 2% eosin  95% ethanol  *d*H_2_O  3% H_2_SO_4_  tap water  *d*H_2_O | 5 min  15 min  5 min  60°C, 1.5 h  30 s  5 min | – Good clearing effect, but in some areas unwanted effect of light reflection. |
|  | dehydration II  clearing | |  |
| 10. | 95% H_2_SO_4_  95% ethanol  *d*H_2_O  3% H_2_SO_4_  tap water  *d*H_2_O | 5 min  15 min  5 min  60°C, 1.5 h  30 s  5 min | – The seed coat could be easily removed.  – The tissue transparency was improved.  – The prolonged incubation time in 100% ethanol (24 h) and pure methyl salicylate (at least 24 h) resulted in better infiltration of the clearing solution, especially for seed development stages. |
|  | dehydration III^i^  clearing II^j^ | |  |

^a^ Ex. No. 1–9: ovules in pre- and post-fertilization stages; Ex. No. 10: ovules in post-fertilization stages.

^b^ All the steps were performed at room temperature, unless otherwise specified.

^c^ Dehydration = ethanol: 70% (15 min); 95%, 100% (1 h each).

^d^ Clearing = 100% ethanol : methyl salicylate, in proportions of 3:1, 1:1, 1:3 (2 h each change, with 3 min vortexing before and after treatment; after 1 h of each change, vacuum treatment for 5 min was applied); pure methyl salicylate (at least 24 h).

^e^ Punctures were performed directly on slide under control of stereomicroscope, in methyl salicylate solution, with a syringe needle nearby the embryo sac.

^f^ dissolved in 95% ethanol.

^g^ Sulfur water was made with 5 mg mL^-1^ K­_2_S_2_O_5_ in 0.05 M HCl.

^h^ Dehydration II = ethanol: 10%, 30%, 50%, 70% (15 min each); 95%, 100% (1 h each).

^i^ Dehydration III = ethanol: 10%, 30%, 50%, 70% (15 min each); 95% (1 h); 100% (24 h).

^j^ Clearing II = 100% ethanol : methyl salicylate, in proportions of 3:1, 1:1 (2 h each change); 1:3 (24 h) (with 3 min vortexing before and after treatment; after 1 h of each change, vacuum treatment for 5 min was applied); pure methyl salicylate (at least 24 h).
