# Supplementary figures and images for: Refinement of a clearing protocol to study crassinucellate ovules of the sugar beet (Beta vulgaris L., Amaranthaceae)
Source: Plant Methods. 2019 Jul 8;15:71. doi: 10.1186/s13007-019-0452-6 (PMC6613245; doi:10.1186/s13007-019-0452-6)

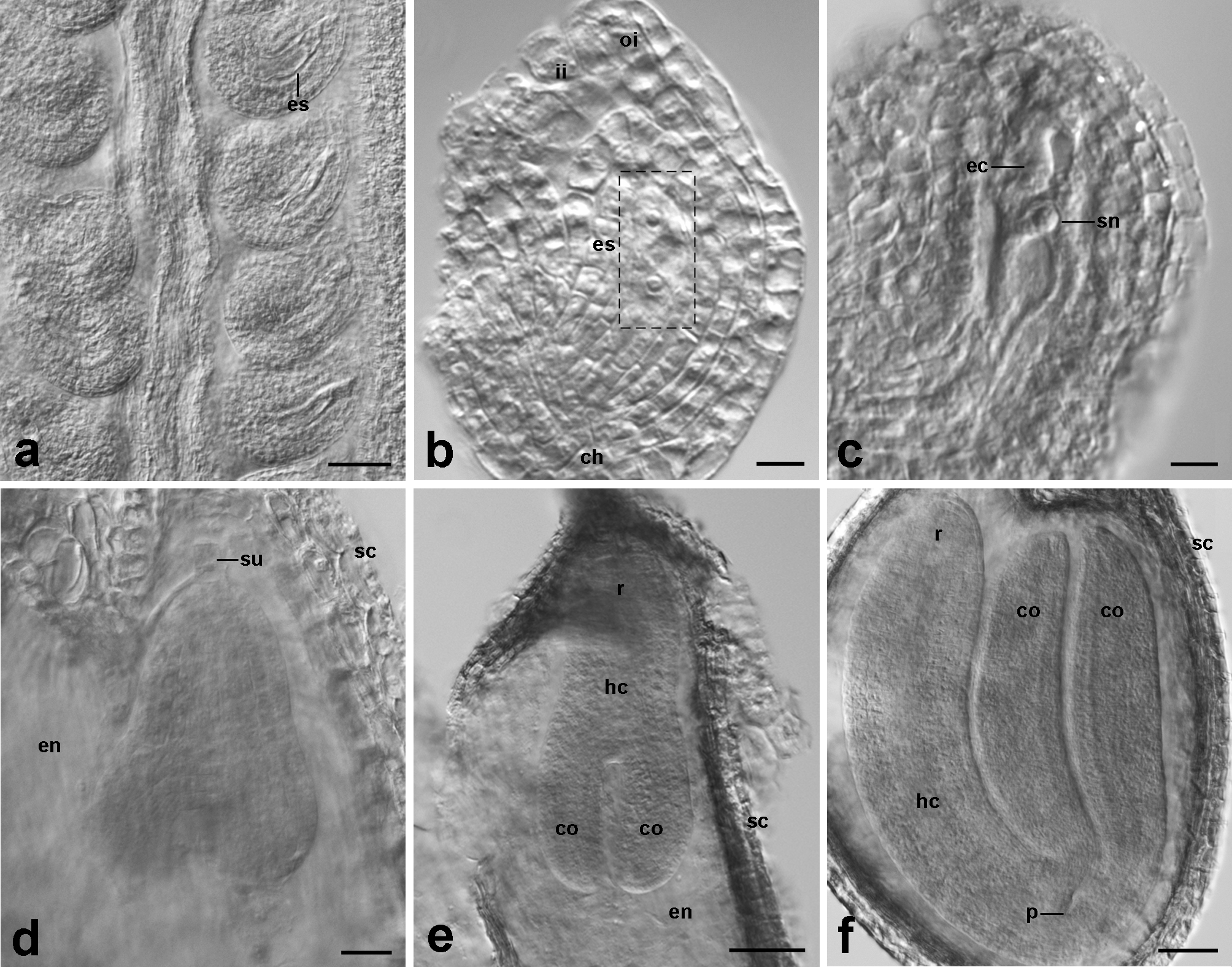

Supplement: Supplementary file 2 — Additional file 2: Fig. S1. Differential interference contrast (DIC) images of Arabidopsis thaliana ovules in subsequent stages of development cleared by the standard procedure with methyl salicylate. (a) Fragment of an ovary with multiple ovules. (b) Young ovule with two-nucleate embryo sac. (c) Ovule with mature embryo sac. (d) Early torpedo-shaped embryo. (e) Late torpedo-shaped embryo. (f) Mature embryo. ch, chalazal pole of the ovule; co, cotyledon; ec, egg cell; en, cellular endosperm; ep, epidermis; es, embryo sac; hc, hypocotyl; ii, inner integument; oi, outer integument; p, plumule; r, radicle; sc, seed coat; sn, secondary nucleus of the central cell; su, suspensor. Scale bars: 10 µm (b, c), 20 µm (d), 50 µm (a, e, f). [file 13007_2019_452_MOESM2_ESM.png]

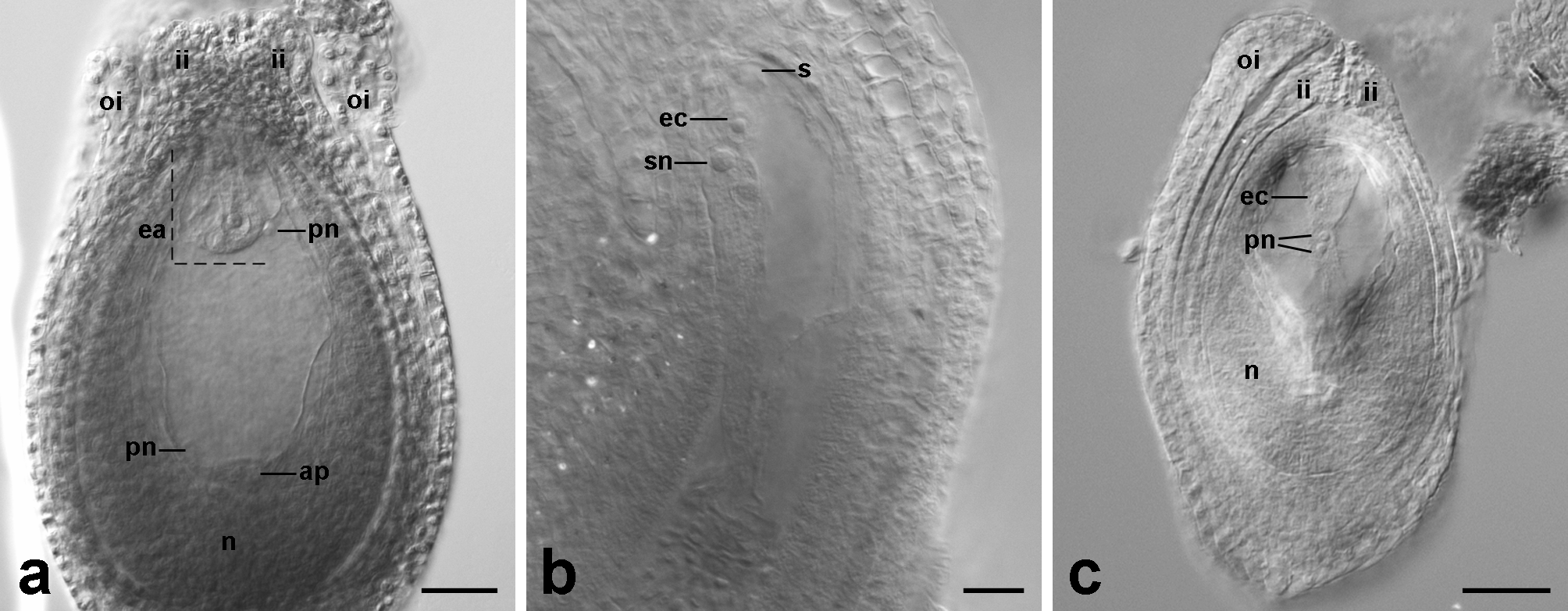

Supplement: Supplementary file 3 — Additional file 3: Fig. S2. Differential interference contrast (DIC) images of Armeria maritima (a), Biscutella laevigata (b), and Viola banksii (c) ovules cleared by the standard procedure with methyl salicylate. ap, antipodal cells; ea, egg apparatus; ec, egg cell; ii, inner integument; n, nucellus; oi, outer integument; pn, polar nucleus; s, synergid; sn, secondary nucleus of the central cell. Scale bars: 20 µm (b), 50 µm (a, c). [file 13007_2019_452_MOESM3_ESM.png]
